# Analysis of cystic fibrosis gene mutations in children with cystic fibrosis and in 964 infertile couples within the region of Basilicata, Italy: a research study

**DOI:** 10.1186/1752-1947-8-339

**Published:** 2014-10-10

**Authors:** Domenico Dell’Edera, Michele Benedetto, Gemma Gadaleta, Domenico Carone, Donatello Salvatore, Antonella Angione, Massimiliano Gallo, Michele Milo, Maria Laura Pisaturo, Giuseppe Di Pierro, Eleonora Mazzone, Annunziata Anna Epifania

**Affiliations:** 1 Unit of Cytogenetics and Molecular Genetics, ‘Maddonna delle Grazie Hospital’, street Cattedra Ambulante, 75100 Matera, Italy; 2 Department of Biochemistry and Molecular Biology Ernesto Quagliariello, University of Bari, street Orabona 4, 70125 Bari, Italy; 3 Center of Reproduction and Andrology (CREA), street Scoglio del Tonno 79/81, 74100 Taranto, Italy; 4 Cystic Fibrosis Centre, AOR San Carlo Hospital, street Potito Petrone 1, 85100 Potenza, Italy; 5 Regional Center of Pharmacovigilance, Basilicata Region, street Potito Petrone 1, 85100 Potenza, Italy; 6 Unit of Obstetrics and Gynecology, AOR San Carlo Hospital, street Potito Petrone 1, 85100 Potenza, Italy; 7 Department of Surgical Science, University of Parma, Parma Hospital, street Gramsci 14, 43100 Parma, Italy; 8 Unit of Clinical Chemistry, ‘Madonna delle Grazie Hospital’, street Cattedra Ambulante, 75100 Matera, Italy

**Keywords:** Cystic fibrosis, Cystic fibrosis conductance transmembrane regulator, Screening in infertile couples, Mutation analysis, Polymerase Chain Reaction

## Abstract

**Introduction:**

Cystic fibrosis is the most common autosomal recessive genetic disease in the Caucasian population. Extending knowledge about the molecular pathology on the one hand allows better delineation of the mutations in the *CFTR* gene and the other to dramatically increase the predictive power of molecular testing.

**Methods:**

This study reports the results of a molecular screening of cystic fibrosis using DNA samples of patients enrolled from January 2009 to December 2013. Patients were referred to our laboratory for cystic fibrosis screening for infertile couples. In addition, we identified the gene mutations present in 76 patients affected by cystic fibrosis in the pediatric population of Basilicata.

**Results:**

In the 964 infertile couples examined, 132 subjects (69 women and 63 men) resulted heterozygous for one of the *CFTR* mutations, with a recurrence of carriers of 6.85%. The recurrence of carriers in infertile couples is significantly higher from the hypothetical value of the general population (4%).

**Conclusions:**

This study shows that in the Basilicata region of Italy the *CFTR* phenotype is caused by a small number of mutations.

Our aim is to develop a kit able to detect not less than 96% of *CTFR* gene mutations so that the relative risk for screened couples is superimposable with respect to the general population.

## Introduction

Cystic fibrosis (CF) is the most common autosomal recessive genetic disease for the Caucasian (white) population. In Italy, the disease occurs in 1/2500 to 1/3000 Caucasian newborns, with a carrier incidence ranging from 1/26 to 1/30 in the general population [1,2].

CF is a complex multisystem disease related to the buildup of thick, sticky mucus that can damage many of the body's organs (epithelia of the respiratory tract, exocrine pancreas, intestine, male genital tract, hepatobiliary system, and exocrine sweat glands). The pulmonary disease is present in 90% of patients and it is the major cause of morbidity and mortality in CF.

The *CFTR* gene provides instructions for making a glycoprotein called cystic fibrosis transmembrane conductance regulator (1,480 amino acid residues). This protein works as a channel across the membrane of cells producing mucus, sweat, saliva, tears, and digestive enzymes. The channel negatively transports charged particles called chloride ions into and out of the cells. The transport of chloride ions helps control the movement of water in tissues, which is necessary for the production of thin, freely flowing mucus. Mucus is a slippery substance that lubricates and protects the lining of the airways, digestive system, reproductive system and other organs and tissues [3]. In particular, more than 95% of men with CF are infertile as a result of azoospermia caused by congenital bilateral agenesis of the vas deferens (CBAVD); it occurs in men without pulmonary or gastrointestinal manifestations of CF.

Cystic fibrosis is caused by mutations in the CFTR gene (cystic fibrosis transmembrane regulator), detected for the first time in 1989. The *CFTR* gene is located on the long (q) arm of chromosome 7 (7q31.2) [4,5].

More than 1800 mutations in the CFTR gene have been identified [6]; many of which are so rare as to be called ‘private’ as they are only present within individual families. Moreover, the *CFTR* mutation detection rate varies by test method and ethnic background. The most common mutation, called F508del, is a deletion of one amino acid at position 508 in the CFTR protein. The resulting abnormal channel breaks down shortly after it is made, so it never reaches the cell membrane to transport chloride ions. The F508del mutation, accounts for two-thirds of all cystic fibrosis alleles worldwide. This mutation is particularly frequent in people of northern European ancestry (70% in Anglo-Saxon countries and 50% in the Mediterranean area).

The phenotypic variability is determined by the heterogeneity of mutations in the *CFTR* gene, but also by many other factors, such as modifier genes, epigenetic regulation, environment and timeliness in therapy [7,8].

Carrier screening for cystic fibrosis involves analysis for common mutations in the *CFTR* gene from people with no personal history, or family history, of the disease.

This analysis shows whether a person is a carrier, at risk (one in four) of having a baby with cystic fibrosis if their partner is also a carrier. Carrier screening has been recommended by the American College of Medical Genetics and American College of Obstetricians and Gynecologists (ACMG/ACOG) [9] and the Human Genetics Society of Australasia (HGSA) [10] and has been established in the USA, Australia, and parts of Europe [11]. Accurate identification of CF mutations results in more applicable programs for prevention, diagnosis, and treatment of CF. In a study carried out in some areas of Northern Italy, carrier screening was associated with a decrease in the incidence of CF [12].

Previous reports about CF patients born in the Basilicata region (southern Italy) have identified types and incidence of the most common mutations of the *CFTR* gene [13,14].

In this study are reported the results of a molecular screening of CF in patients enrolled from January 2009 to October 2013 using DNA samples. Patients were referred to our laboratory for CF screening for infertile couples [15]. In addition, we identified the gene mutations present in patients affected by cystic fibrosis in the pediatric population of Basilicata.

In this way the incidence and typology of *CFTR* mutations in Basilicata were characterized and related to previous reports on CF patients born in the same area.

## Results

In the 964 infertile couples examined, 132 subjects (69 women and 63 men) were heterozygous for one of the mutations of *CFTR*, with a carrier occurrence of 6.85% (Table 1). The carrier occurrence in infertile couples is significantly higher than the hypothetical value of the general population (4%). The statistical analysis was performed according to the ‘hypothesis test for a proportion’. Our data indicated an expectation of 6.85% with a sample of 1,928 subjects. The Z-test revealed a value of 6.38% with a *P* value <0.0001. Nevertheless, mutation I148T, at first classified as a mutation, now is considered an ‘innocent’ polymorphism, able to be pathogenetic only if present in the same gene (on the same chromosome) with another polymorphism (3199del6). Hence it is necessary, in presence of polymorphism I148T, to detect the presence of polymorphism 3199del6. The polymorphism 3199del6 was not detected in all patients with the I148T variant.

**Table 1:** **Number of subjects tested who were carriers of the *cystic fibrosis transmembrane regulator *** **gene**

**Mutation**	**Men**	**Women**	**Total**
G551D	1	2	3
R553X	0	1	1
F508del	35	32	67
N1303K	7	8	15
I148T	4	9	13
G542X	3	6	9
DI507	2	0	2
L1077P	0	2	2
D1152H	1	6	7
W1282X	2	0	2
2183 AA>G	3	0	3
1259insA	0	1	1
4016insT	1	0	1
I507del	1	0	1
2789+5G>A	1	0	1
4382delA	0	2	2
G1244E	1	0	1
621+3A>G	1	0	1
Total	63	69	132

Among the infertile couples, three men, two of whom were brothers, were azoospermic with CBAVD. The molecular analysis of the *CFTR* gene revealed that the two brothers, aged 26 and 29 years old respectively, were both ‘compound heterozygotes G542X/D1152H’, while the third one, aged 37 years old, was ‘compound heterozygotes G542X/E831X’.

**Figure 1:**
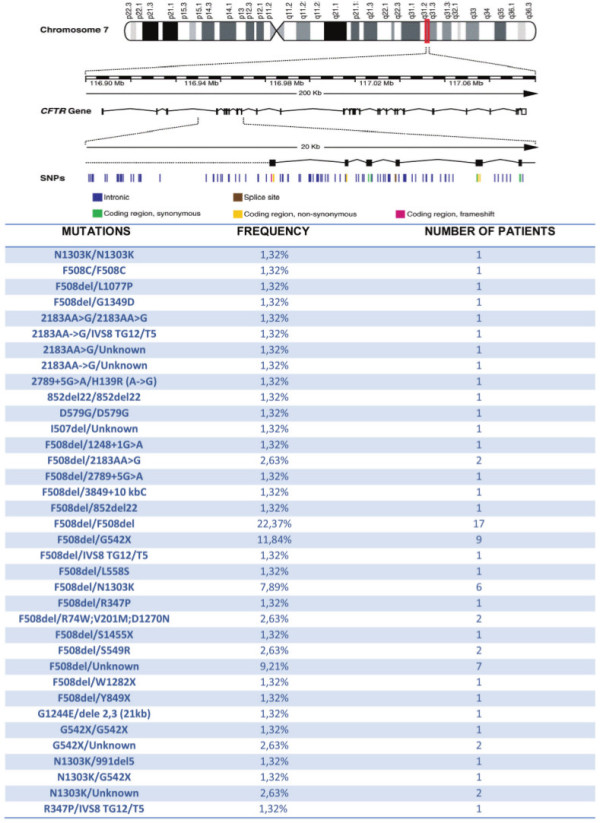
**76 patients with cystic fibrosis and positive sweat test, all have two genes mutated.** SNPs: Single nucleotide polymorphism.

With regard to the 76 patients with cystic fibrosis and a positive sweat test, all have two *CFTR*-mutated genes (Figure 1).

## Discussion

The aim of this study was to characterize, at molecular level, the most common *CFTR* mutations in the population of the Basilicata region. We have defined the type and frequency of mutations found and put them in comparison with data from previous studies (Table 2) [13,14]. In this study, we detected mutations that had not been revealed in the two previous studies [13,14]. In infertile couples from Basilicata, the occurrence of CF carriers is significantly higher than in the general population (6.85% vs. 4%). It was not clear if a screening for the general population would be viable or effective, so we invited the carriers in the families of affected people to participate in the research.

**Table 2:** Comparison between the results obtained in this study and those obtained in a previous study

	**Castaldo *et al*.**[14]	**Mutations observed in the present study**
**F508del**	55.8% (29)	48.62% (141)
**N1303K**	3.8% (2)	9.31% (27)
**G542X**	3.8% (2)	8.96% (26)
**W1282X**	3.8% (2)	1.03% (3)
**2183AA>G**	5.8% (3)	2.76% (8)
**R1162X**	0	0
**1717-1G>A**	1.9% (1)	0
**T338I**	0	0
**R347P**	0	0.69% (2)
**711+5G>A**	0	0
**852del22**	5.8% (3)	1.03% (3)
**4382delA**	0	0.69% (2)
**1259insA**	0	0.34% (1)
**4016insT**	0	0.34% (1)
**R553X**	0	0.34% (1)
**R1158X**	0	0
**L1077P**	0	1.03% (3)
**I502T**	0	0
**3849+10kbC>T**	1.9% (1)	0.34% (1)
**D579G**	0	0.69% (2)
**G1244E**	3.8% (2)	0
**G1349D**	0	0.34% (1)
**2789+5G>A**	0	1.03% (3)
**711+1G>T**	0	0
**L1065P**	0	0
**2522insC**	0	0
**E585X**	0	0
**G85E**	0	0
**G178R**	0	0
**D1152H**	0	3.10% (9)
**I148T-3195del6**	0	0
**I148T (alone)**	0	4.48% (13)
**R334W**	0	0
**DI507**	0	0.69% (2)
**I1005R**	0	0
**3272-26A>G**	0	0
**2711delT**	0	0
**L558S**	1.9% (1)	0.34% (1)
**W1063X**	0	0
**D110H**	0	0
**S549R (A>C)**	1.9% (1)	0.69% (2)
**2184insA**	0	0
**3131del22**	0	0
**R709N**	0	0
**A349V**	0	0
**4015insA**	0	0
**Y849X**	1.9% (1)	0.34% (1)
**G551D**	0	1.03% (3)
**621+3A>G**	0	0.34% (1)
**E831X**	0	0
**I507del**	0	0.69% (2)
**IVS8 TG12/t5**	0	1.03% (3)
**H139R (A->G)**	0	0.34% (1)
**1248+1G>A**	0	0.34% (1)
**R74W;V201M;D1270N**	0	0.69% (2)
**S1455X**	0	0.34% (1)
**dele 2,3 (21kb)**	0	0.34% (1)
**991del5**	0	0.34% (1)
**UNKNOWN**	7 %(4)	4.83% (14)
**F508C**	0	0.69% (2)
**TOTAL**	**52**	**290**

In CF, contrary to other genetic diseases (for example beta thalassemia), the identification of carriers is possible only through molecular research of *CFTR* mutations. A recent report has shown the costs of managing CF for the Italian National Health System [16]. The results reports a high cost for the chronic and evolutive aspects of the disease. As mentioned before, molecular screening of CF is highly recommended in the USA by the National Institutes of Health Consensus Development Conference Statement on genetic testing for cystic fibrosis [17]. The target of the screening could be men and women of reproductive age in the periconceptional period. A European consensus conference in 2009 has concluded that for complete information for couples, each national health system could implement a screening program. In Italy, the region of Veneto started a complete screening program for the general population some years ago. The results have shown a significant reduction of the incidence of the disease. More recently, an experimental periconceptional program of CF and thalassemia screening allowed for the identification of 94% of *CFTR* gene mutations, with respect to 80% in the rest of Italy. Moreover, this screening program involved a high number of couples [18].

The success of a genetic screening program is mainly the outcome of educational support informing the population about the disease, thereby offering the opportunity to make an informed decision about reproduction.

In this study, the aim was to establish the *a priori* risk of CF carrier status in the Basilicata population and to offer a reliable screening test to couples planning a pregnancy, allowing them to make informed decisions.

## Conclusions

This study shows that, in the Basilicata region (Italy), it was observed that the *CFTR* phenotype is caused by a small number of mutations.

The knowledge of the frequency of mutations prevalent in *CFTR* genes in the province of Matera allows development of kits for their detection.

Our aim is to develop a kit able to detect not less than 96% of *CTFR* gene mutations so that the relative risk for the couples screened is superimposable with respect to the general population.

Extending knowledge about the molecular pathology, on the one hand, allows better delineation of the mutations in the *CFTR* gene, and on the other, dramatically increases the predictive power of molecular testing.

## Methods

In this study, we investigated 964 infertile couples (1.928 subjects examined; 3.856 alleles studied) born and living in Basilicata, from 2009 to December 2013 (five years). Moreover, we evaluated gene mutation of *CFTR* in 76 children born in Basilicata (152 CF alleles examined) with a positive sweat test (>60mmol/L). Written informed consent was obtained from the subjects examined for publication of this study in compliance with the Helsinki Declaration.

We applied the following tests to each patient after a venous blood sample was collected (in EDTA-K3).

Molecular analysis of the *CFTR* gene was performed following these steps:

– DNA isolation, starting from 25μl of blood, using the Promega extraction kit (DNA IQ™ System, cod.C6701; Promega Italy S.r.l., Milan, Italy).

– Polymerase chain reaction (PCR) and reverse hybridization. The procedure includes two steps: PCR amplification using biotinylated primers and hybridization of amplification products to a test strip containing allele-specific oligonucleotide probes immobilized as an array of parallel lines. Bound biotinylated sequences are detected using streptavidin-alkaline phosphatase and color substrates. The amplification and the reverse hybridization on a strip were obtained with the use of commercial kits produced by Nuclear Laser Medicine S.r.l., Settala (MI), Italy (cod. AC023/AC025 and AC089): genetic tests aimed at checking 60 mutations in the *CFTR* genes. The mutations analyzed are listed in Table 3. The test has a sensitivity and a specificity of more than 99%. With a direct analysis of 60 mutations of the *CTFR* gene, with reverse dot blot, it is possible to detect 90% of the most common CF alleles in Southern Italy (the regions of Campania, Puglia, Basilicata and Molise).

– The patients who tested negative or with a single mutation detected by reverse dot blot and with a clinical suspicion of atypical cystic fibrosis were analyzed with a complete scanning of the codificant region, through amplification and direct sequencing of 27 exones of the *CFTR* gene. In patients negative for reverse dot blot and in whom there were no clinical signs of cystic fibrosis, sequencing of the *CFTR* gene was not necessary. DNA sequencing is not essential, since the detection of innocent polymorphisms is not important to control the disease. The sequence of oligonucleotides for each exone, with the annealing temperatures (T°A) and the length in base pair (bp) of the amplified product, are reported in Table 4. The amplification conditions for the 27 exones change according to the annealing temperature (T°A.), which depends on the oligonucleotides used, which in turn are specific for analysis of each exone. The amplification report is shown in Table 5.

**Table 3:** **List of 60 mutations in the *cystic fibrosis transmembrane regulator *** **gene (specificity 100%)**

F508del	I507del	F508C	621+1G>T	D110H	E585X	G1349D
I502T	1706del17	1677delTA	R117H	H139R	1898+1G>A	4015delA
G542X	1717-1G>A	Q552X	852del22	G178R	1898+3A>G	
G551D	S549R(A>C)	2183AA>G	T338I	991del5	1898+5G>T	
N1303K	4016insT	3849+10kb C>T	R347P	R334W	2184insA	
G85E	711+5G>A	711+1G>T	1259insA	R347H	2522insC	
2789+5G>A	W1282X	G1244E	R1066H	R352Q	3120+1G>A	
I148T	3199del6	S912X	R1158X	1717-8G>A	R1066C	
R1162X	4382delA	D1152H	L1077P	D579G	3272-26A>G	
L1065P	R553X	PoliT: 5T, 7T, 9T		1874insT	3659delC	

**Table 4:** Sequence of oligonucleotides for each exon, with the annealing temperatures (T°A) and the length in base pair (bp) of the amplified product

**Exon**	**Sequence of primers**	**T°A**	**bp**
1	GAGAAAGCCGCTAGAGCAAA(CF1F)	55°C	394
	TCCTTTACCCCAAACCCAAC(CF1R)		
2	TCCAAATCTGTATGGAGACCA(CF2F)	55°C	603
	TCAGTGTGAAAATGAGATGTTCC(CF2R)		
3	TCTGGCTGAGTGTTTGGTGT(CF3F)	55°C	399
	TTTGGAGTTGGATTCATCCTTT(CF3R)		
4	AAACTTGTCTCCCACTGTTGC(CF4F)	55°C	453
	GGCCTGTGCAAGGAAGTATT(CF4R)		
5	GTGCCTAGATGCTGGGAAAT(CF5F)	55°C	393
	AAAACTCCGCCTTTCCAGTT(CF5R)		
6a	TGCTATGTGCTCCATGTAATGA(CF6AF)	55°C	415
	TGCATAGAGCAGTCCTGGTT(CF6AR)		
6b	TGCCCATCTGTTGAATAAAAG(CF6BF)	55°C	411
	CCCATGAAAGTGAATTTGTGC(CF6BR)		
7	TTCCATTCCAAGATCCCTGA(CF7F)	55°C	404
	GCACATTTTTGCAAAGTTCA(CF7F)		
8	GAATCCTAGTGCTTGGCAAAT(CF8F)	55°C	404
	GATCCTCCTTCCAGTTCTACCA(CF8R)		
9	GGCCATGTGCTTTTCAAACT(CF9F)	55°C	389
	CTCCAAAAATACCTTCCAGCA(CF9R)		
10	TGAATCCTGAGCGTGATTTG(CF10F)	55°C	435
	TTCATGTGTTTGCAAGCTTCTT(CF10R)		
11	GAAGGAAGATGTGCCTTTCAA(CF11F)	55°C	395
	CCAAGATACGGGCACAGATT(CF11R)		
12	TCAGTGAATCGATGTGGTGAC(CF12F)	55°C	419
	ATGAGGCGGTGAGAAAAGGT(CF12R)		
13-1	TCATGCTATCAGAATTCACAAGG(CF13F1)	56°C	575
	GGGAGTCTTTTGCACAATGG(CF13R1)		
13-2	CTGGAGAGTTTGGGGAAAAA(CF13F2)	56°C	449
	AAATACCCCCAAGCGATGTA(CF13R2)		
14a	CAATGGTGGCATGAACTGT(CF14AF)	55°C	437
	GTGGTTCTACTTGTTGATTTTTCAG(CF14AR)		
14b	TGGCTTTCTTGTGAGGTTCA(CF4BF)	55°C	446
	TGCTTGGGAGAAATGAAACA(CF14BR)		
15	GTCGCCAAATAACGATTTCC(CF15F)	55°C	406
	AGGTTCAACAAAGGGCACAT(CF15R)		
16	TTTGGGTTCTGAATGCGTCT(CF16F)	55°C	388
	GGCCAGGTAAGCAGTTCTGA(CF16R)		
17a	CTCACCAACATGTTTTCTTTGA(CF17AF)	55°C	399
	CCAAAATGAAGTCACATGGTCA(CF17AR)		
17b	GAATGGCACCAGTGTGAAAA(CF17BF)	55°C	682
	CAATCTGTGTGCATCGGTTT(CF17BR)		
18	TGTGCCCTAGGAGAAGTGTG(CF18F)	55°C	335
	TGACAGATACACAGTGACCCTCA(CF18R)		
19	GCCCGACAAATAACCAAGTG(CF19F)	55°C	399
	GCAAGCAGTGTTCAAATCTCA(CF19R)		
20	CCAATTCCTTATGCCCAGTT(CF20F)	55°C	408
	TGGCTAAGTCCTTTTGCTCA(CF20R)		
21	TGATGGTAAGTACATGGGTGTTTC(CF21F)	57°C	578
	GGAGCCATACCAGTGAGGAG(CF21R)		
22	TCAAATGGTGGCAGGTAGTG(CF22F)	55°C	382
	TCACCATGAAGCAGGCATAA(CF22R)		
23	CCCATGGTTGAAAAGCTGAT(CF23F)	55°C	417
	TGAGTAAAGCTGGATGGCTG(CF23R)		
24	GCCTTCTGTCCCAGATCTCA(CF24F)	60°C	362
	GAGCAAATGTCCCATGTCAA(CF24R)		

**Table 5:** Amplification report

**Temperature**	**Time**	**Cycles**
95°C	5’	1
95°C	30”	35
T° A	30”	
72°C	20”	
72°C	5’	1

### Consent

Written informed consent was obtained from all of the patients (including legal guardians of the children) for publication of this case report and accompanying images. A copy of the written consent is available for review by the Editor-in-Chief of this journal. Our local institutional Ethics Committee approved this study.

## Competing interests

The authors declare that they have no competing interests.

## Authors’ contributions

DD made substantial contributions to conception and design. MB, MM, AA and MG contributed to the acquisition, analysis and interpretation of data. DS, AAE and GG were involved in drafting the manuscript. DC, MLP, GD and EM gave final approval of the version to be published. All authors read and approved the final manuscript.
